# Supplementary material for: High entropy engineered polymer blends with enhanced dielectric properties and high temperature stability
Source: Nat Commun. 2025 Oct 13;16:9056. doi: 10.1038/s41467-025-63248-x (PMC12518587; doi:10.1038/s41467-025-63248-x)
Supplement: Supplementary file 1 — Supplementary Information [file 41467_2025_63248_MOESM1_ESM.pdf]

Supplementary Information

High Entropy Engineered Polymer Blends with Enhanced Dielectric Properties and High Temperature Stability

Xin Qi[1], Xuankai Huang[2], Nasima Kanwal[2], Bijoy Das[1], Anthony Phillips[2], Dimitrios Papageorgiou[1], Haixue Yan[1], Emiliano Bilotti[3], and Michael John Reece[1]*

[1] School of Engineering and Materials Science
Queen Mary University of London
Mile End Road, London E1 4NS, UK

[2] School of Physical and Chemical Sciences
Queen Mary University of London
Mile End Road, London E1 4NS, UK

[3] Department of Aeronautics
Imperial College London
Exhibition Road, London SW7 2AZ, UK

*Corresponding author:
Michael John Reece (m.j.reece@qmul.ac.uk)

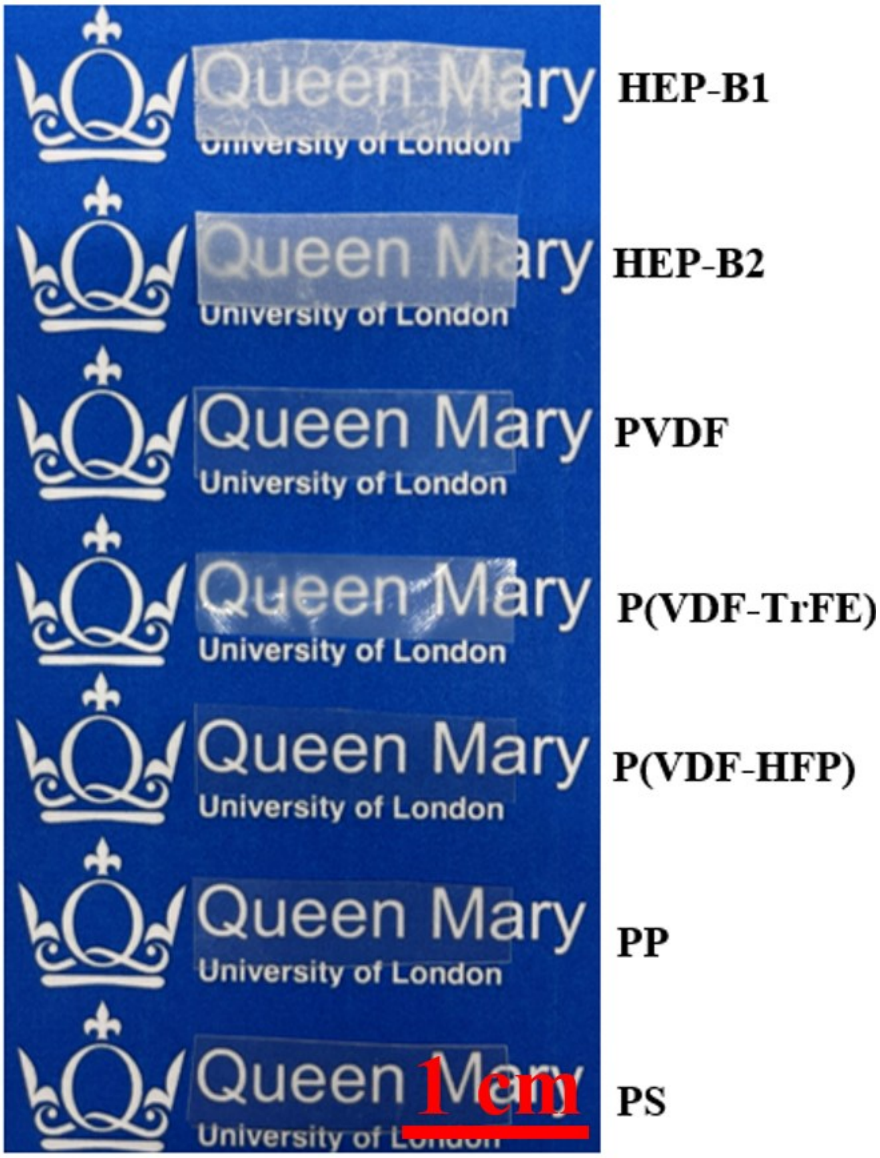

Figure S1. Optical images of polymer films.

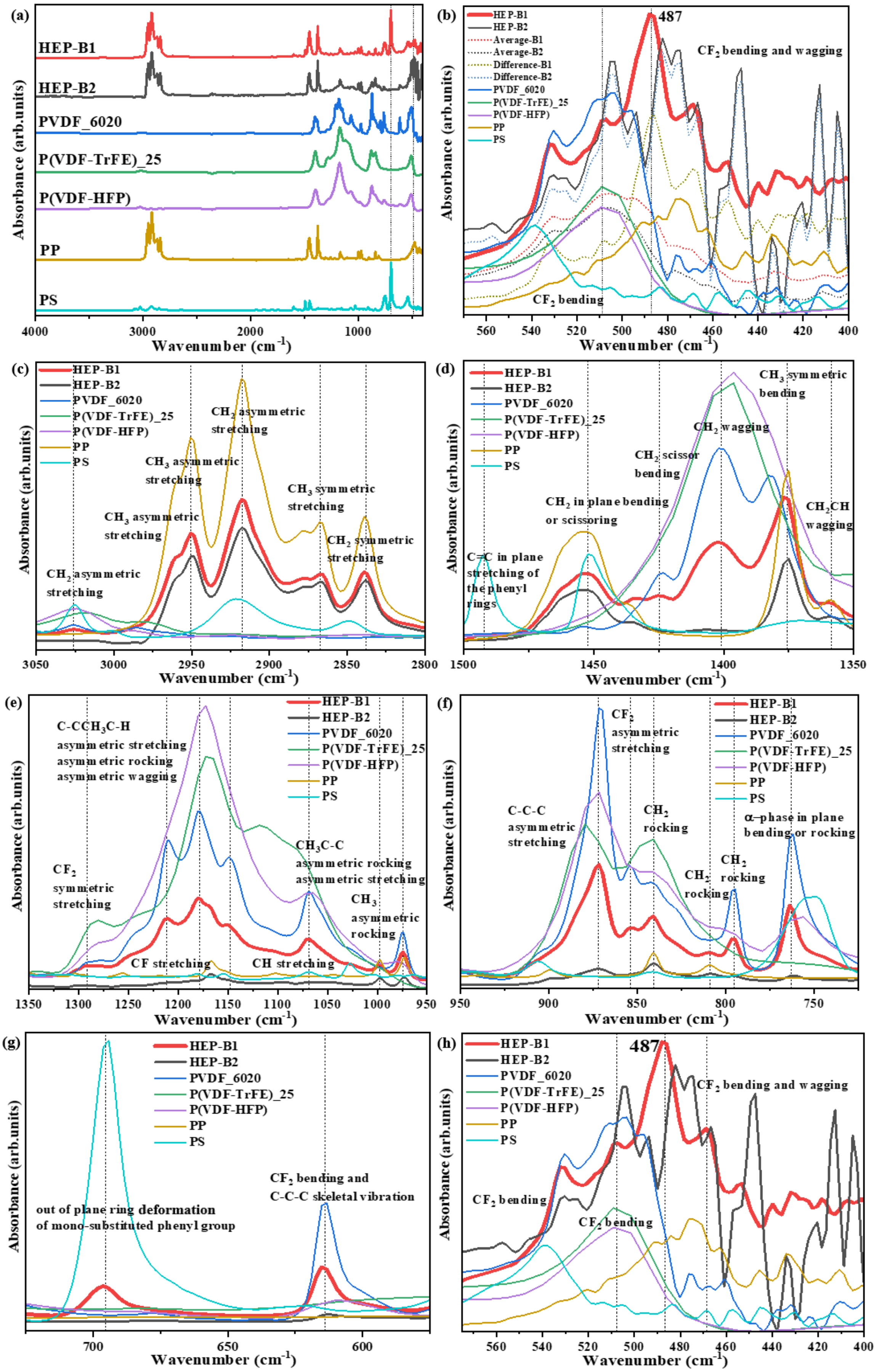

Figure S2. (a)-(h) FTIR of HEP-B1, HEP-B2 polymer blends and individual polymers.

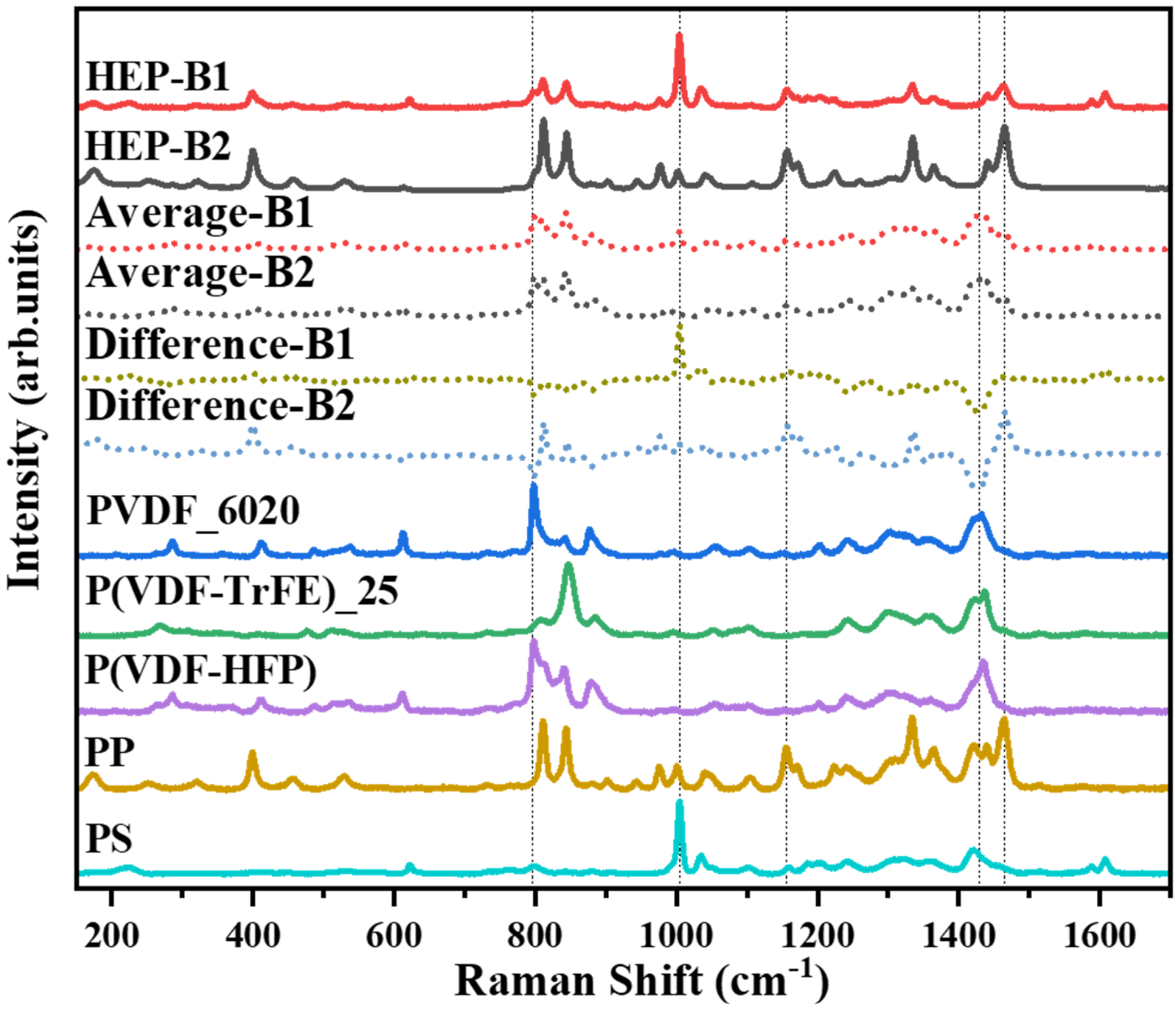

Figure S3. Raman data of HEP-B1, HEP-B2 polymer blends and individual polymers.

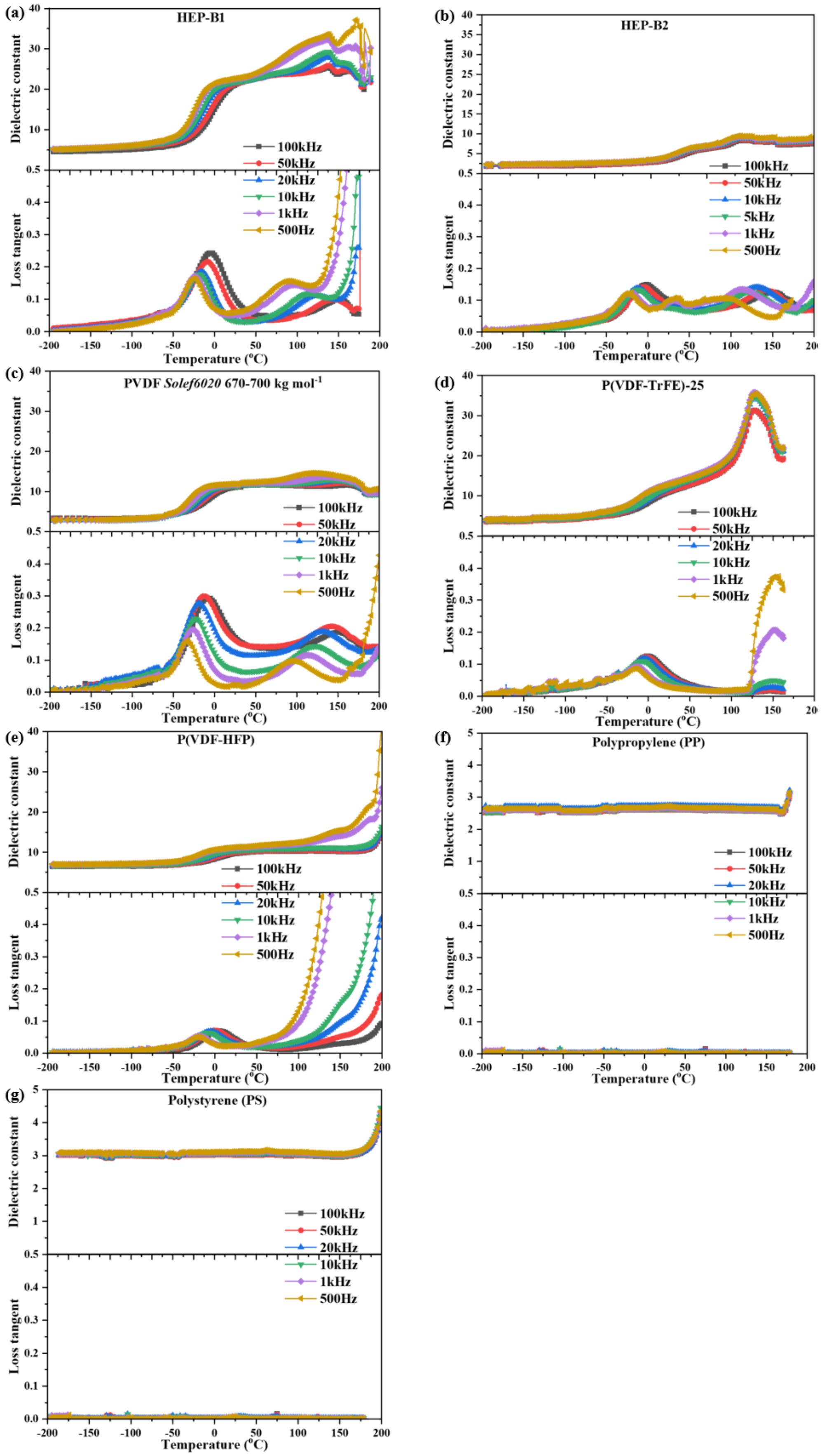

Figure S4. Temperature dependence of dielectric properties of HEP-B1 and HEP-B2 polymer blends and individual polymers for a range of different frequencies.
